# The Allele Catalog Tool: a web-based interactive tool for allele discovery and analysis

**DOI:** 10.1186/s12864-023-09161-3

**Published:** 2023-03-10

**Authors:** Yen On Chan, Nicholas Dietz, Shuai Zeng, Juexin Wang, Sherry Flint-Garcia, M. Nancy Salazar-Vidal, Mária Škrabišová, Kristin Bilyeu, Trupti Joshi

**Affiliations:** 1 MU Institute for Data Science and Informatics, University of Missouri-Columbia, Columbia, MO USA; 2 Christopher S. Bond Life Sciences Center, University of Missouri-Columbia, Columbia, MO USA; 3 Division of Plant Science and Technology, University of Missouri-Columbia, Columbia, MO USA; 4 Department of Electrical Engineering and Computer Science, University of Missouri-Columbia, Columbia, MO USA; 5 United States Department of Agriculture-Agricultural Research Service, Plant Genetics Research Unit, Columbia, MO USA; 6 Department of Evolution and Ecology, University of California-Davis, Davis, CA USA; 7 Department of Biochemistry, Faculty of Science, Palacky University in Olomouc, Olomouc, Czech Republic; 8 Department of Health Management and Informatics, University of Missouri-Columbia, Columbia, MO USA

**Keywords:** Variant Calling Pipeline, Allele Catalog Pipeline, Allele Catalog Tool, Alleles in Gene, Data Visualization

## Abstract

**Background:**

The advancement of sequencing technologies today has made a plethora of whole-genome re-sequenced (WGRS) data publicly available. However, research utilizing the WGRS data without further configuration is nearly impossible. To solve this problem, our research group has developed an interactive Allele Catalog Tool to enable researchers to explore the coding region allelic variation present in over 1,000 re-sequenced accessions each for soybean, *Arabidopsis*, and maize.

**Results:**

The Allele Catalog Tool was designed originally with soybean genomic data and resources. The Allele Catalog datasets were generated using our variant calling pipeline (SnakyVC) and the Allele Catalog pipeline (AlleleCatalog). The variant calling pipeline is developed to parallelly process raw sequencing reads to generate the Variant Call Format (VCF) files, and the Allele Catalog pipeline takes VCF files to perform imputations, functional effect predictions, and assemble alleles for each gene to generate curated Allele Catalog datasets. Both pipelines were utilized to generate the data panels (VCF files and Allele Catalog files) in which the accessions of the WGRS datasets were collected from various sources, currently representing over 1,000 diverse accessions for soybean, *Arabidopsis*, and maize individually. The main features of the Allele Catalog Tool include data query, visualization of results, categorical filtering, and download functions. Queries are performed from user input, and results are a tabular format of summary results by categorical description and genotype results of the alleles for each gene. The categorical information is specific to each species; additionally, available detailed meta-information is provided in modal popups. The genotypic information contains the variant positions, reference or alternate genotypes, the functional effect classes, and the amino-acid changes of each accession. Besides that, the results can also be downloaded for other research purposes.

**Conclusions:**

The Allele Catalog Tool is a web-based tool that currently supports three species: soybean, *Arabidopsis*, and maize. The Soybean Allele Catalog Tool is hosted on the SoyKB website (https://soykb.org/SoybeanAlleleCatalogTool/), while the Allele Catalog Tool for *Arabidopsis* and maize is hosted on the KBCommons website (https://kbcommons.org/system/tools/AlleleCatalogTool/Zmays and https://kbcommons.org/system/tools/AlleleCatalogTool/Athaliana). Researchers can use this tool to connect variant alleles of genes with meta-information of species.

## Background

A large amount of publicly accessible whole-genome re-sequenced (WGRS) data has opened up the possibility for researchers to gain more insight into the allelic variations and the potential outcomes of those mutations. Unlike the genome-wide association studies (GWAS) that mainly focus on the single nucleotide polymorphism (SNP) level and phenotypic traits [1], the studies of allelic variations such as SNPs, insertions, and deletions (Indels) that occur at the gene level are required in order to understand the potential functional effects and their impacts on the phenotypes. Therefore, novel bioinformatics approaches, pipelines, and tools are necessary to enable fast and efficient data processing, aggregation, and visualization that can return genotypic information related to allelic variations in genes to researchers and assist them in advancing their research.

Currently, there are many bioinformatics tools that enable visualization of SNPs and Indels, and also provide analytical capabilities. A few examples of such tools are Tassel [2], FlapJack [3], and SNPViz 2.0 [4] which can assist users in understanding complex traits with genotypic data. Tassel is a software that supports a wide range of functionalities such as association analysis, indel analysis, and phenotype-genotype data integration. The alignment viewer, linkage disequilibrium visualization, Manhattan plot, and genetic distance heatmap are some data visualization capabilities in Tassel that are relevant to SNPs, quantitative trait loci (QTL), and GWAS. FlapJack, on the other hand, is a software that is more focused on genotype visualization for users to compare SNPs in different accessions and map accessions to phenotypes or quantitative trait loci data for assessment. Besides that, SNPViz 2.0 is a web tool that focuses on haplotype analysis and genomic variation functions. It provides hierarchical visualization of accessions along with a color map to represent SNPs and indels in a haplotype block.

Although these tools provide visualizations and statistical methods for the analysis of genotypic data, they are still lacking “gene-specific” information such as amino acid changes caused by alternative allelic variations and genotypic data grouping that can reflect differences in the phenotypic information that varies between genes. Typically, from the GWAS analysis, a list of significant SNPs with some associated statistical significance in the form of Manhattan plots are often the output formats produced by GWAS tools such as Tassel and GAPIT3 [5]. From these GWAS hits, researchers often need to map the significant SNPs back to the gene and allele level in order to identify likely gene candidates [6].

To improve candidate gene exploration by emphasizing functional effect mutations, our research group developed the Allele Catalog Tool for allele query and visualization in soybean, *Arabidopsis*, and maize. Moreover, our research group also developed the variant calling pipeline (SnakyVC) and Allele Catalog pipeline (AlleleCatalog) to empower other researchers to develop Allele Catalog data in the necessary format for their own sequenced accession panel. Currently, the Allele Catalog Tool consists of the soybean, *Arabidopsis*, and maize Allele Catalog datasets that were generated by our research group. Users who have genes of interest can bring their gene ID and accession list to the Allele Catalog Tool to perform queries and visualize both the meta-information and genotypic information.

Using the Allele Catalog Tool, it is possible to discover genes with different types of mutations along with the functional effect changes and amino acid changes. Most importantly, similarities and differences in accessions can be uncovered based on the frequency tables in which the frequencies are calculated by grouping the same alleles with functional effects and amino-acid changes in genes. This method can help researchers in understanding the relationships between a genotype set and a group of accessions. With the Allele Catalog Tool, our group believes that the meta-information and genotypic data are more readily accessible and informative, and they assist researchers in making significant progress in their research work.

### Implementation

#### The variant calling pipeline (SnakyVC)

**Fig. 1:**
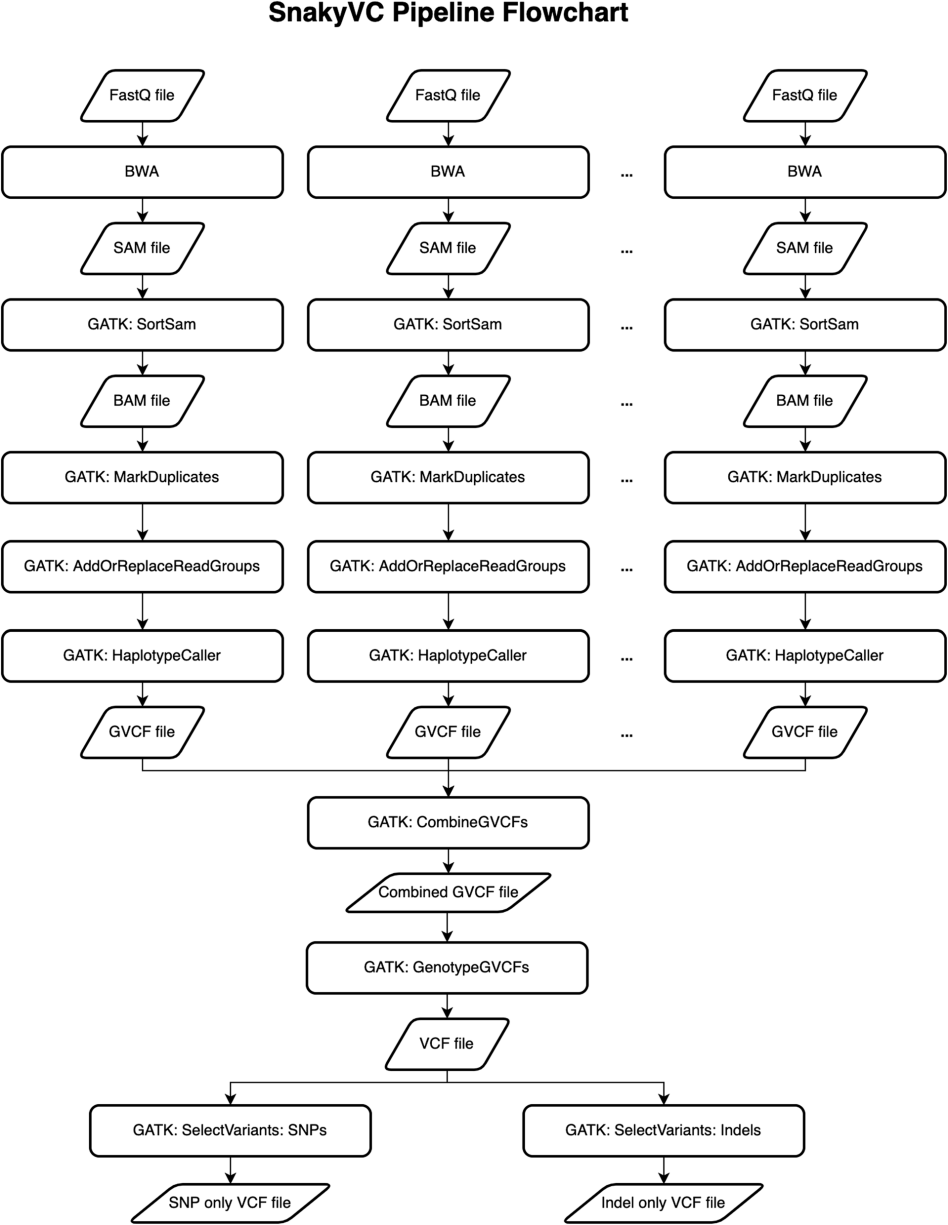
The flowchart of the variant calling pipeline demonstrates each processing step with different tools and commands and the output files that can be generated by the pipeline

The variant calling pipeline (SnakyVC) is a pipeline built for processing the raw sequenced reads to identify the genetic variants in accessions of living organisms. The SnakyVC pipeline is built using Snakemake, which is a bioinformatics workflow management engine [7], by incorporating the Burrows-Wheeler Aligner (BWA) version 0.7.17 [8] and the Genome Analysis Toolkit (GATK) version 4.1.7.0 [9] into one to streamline the processes for different accessions in parallel (Fig. 1).

The inputs of the variant calling pipeline are FASTQ files with raw sequenced reads and a reference genome in FASTA format. The pipeline aligns the raw sequenced reads against the reference genome using the incorporated BWA tool to create the Sequence Alignment/ Map (SAM) files. The SortSam function in the integrated GATK continues the process to sort the SAM files to create Binary Alignment/ Map (BAM) files. The integrated GATK MarkDuplicates function marks duplicated reads in the BAM files then the reads are assigned to read groups by the AddOrReplaceReadGroups function in the integrated GATK. The integrated GATK HaplotypeCaller function takes the final BAM files to re-assemble the haplotypes, call the singular nucleotide polymorphisms (SNPs), insertions, and deletions (Indels), and output the information into Genomic Variant Call Format (GVCF) files. All the GVCF files are merged into one GVCF file using the CombineGVCFs function in the integrated GATK so that the join genotyping process with the integrated GATK GenotypeGVCFs function can be performed to generate the Variant Call Format (VCF) file which has SNPs and Indels of all accessions. The SNPs and Indels in the VCF file can be separated into individual files by using the SelectVariant function in the pipeline as well.

The pipeline is designed to support running on both standalone servers and high-performance computing clusters. Using the pipeline on a standalone server, the running tasks are automatically distributed to available computing cores in the machine while waiting tasks are lining up in a queue. Nonetheless, when the pipeline is utilized on the high-performance computing cluster, the number of running tasks is based on available computing nodes and computing cores. Each running task is executed in a computing node that has available computing cores. To adjust the inputs, outputs, and the number of concurrently running tasks, a configuration file in JavaScript Object Notation (JSON) format is mandatory for the pipeline to function properly. Users can modify the configuration file based on the number of FASTQ samples they have and the specifications of the machine or high-performance computing cluster they are using.

#### The Allele Catalog pipeline (AlleleCatalog)

**Fig. 2:**
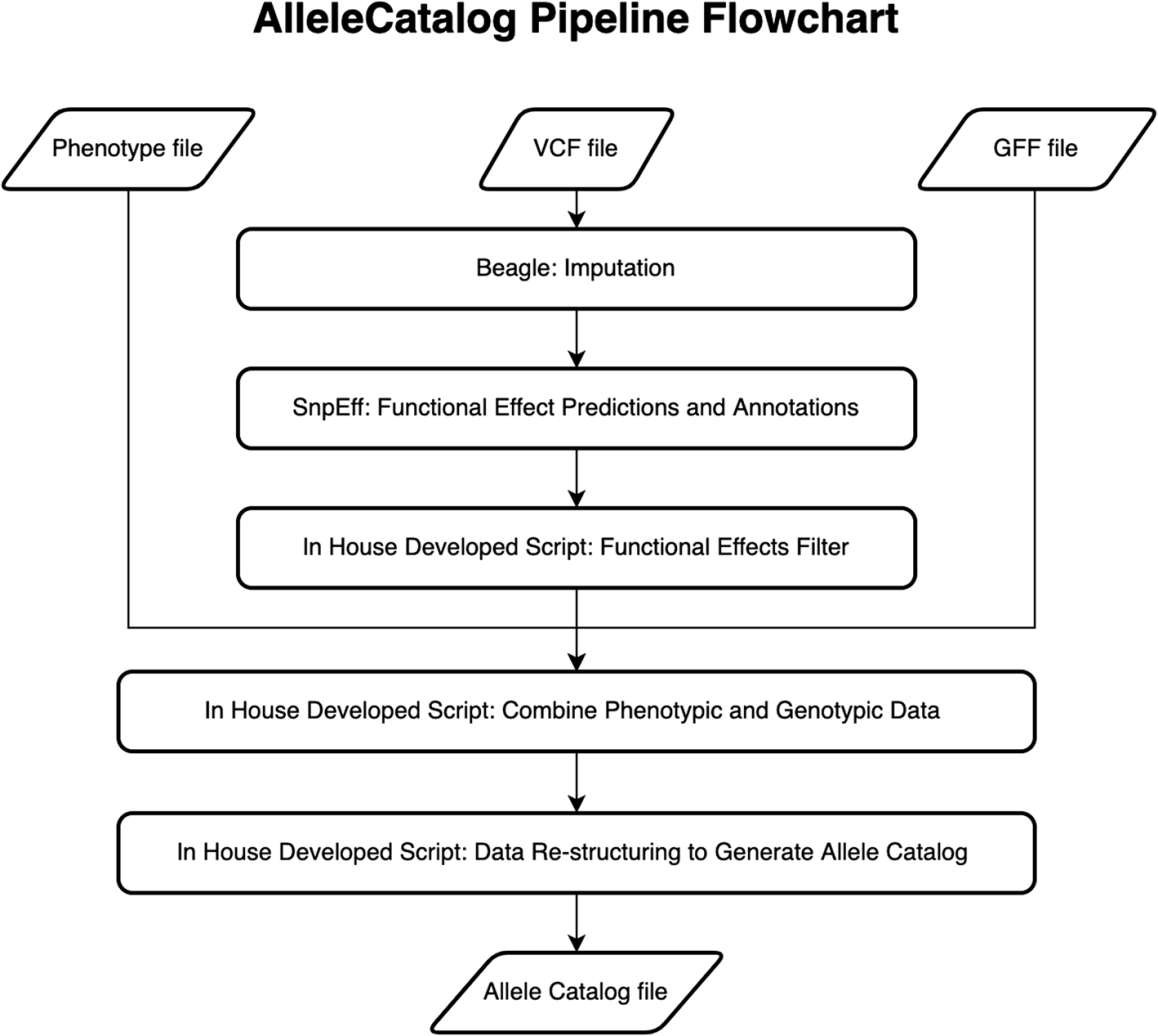
The flowchart of the Allele Catalog pipeline shows the pipeline can automatically run tools and scripts in the processing steps to generate the Allele Catalog datasets

Apart from creating the variant calling pipeline, our research group also created an Allele Catalog pipeline (AlleleCatalog). The purpose of creating the Allele Catalog pipeline is to generate Allele Catalog datasets that connect genes to genetic variants information and meta-information. Similar to the variant calling pipeline, the Allele Catalog pipeline is developed using the Snakemake Bioinformatics workflow management engine. In the Allele Catalog pipeline, the Beagle imputation tool version 5.2 [10], the SnpEff functional effect prediction tool version 5.1 [11], and some of our in-house developed scripts like functional effect extraction script (generate_functional_effect_data.py), imputation information extraction script (generate_imputation_data.py), genotype data extraction script (generate_genotype_data.py), and Allele Catalog data generation script (generate_Allele_Catalog.py) are incorporated into the pipeline to provide parallelizable streamline data analysis and aggregation capabilities to generate the Allele Catalog datasets (Fig. 2).

The Allele Catalog pipeline takes a meta-information file, VCF file, and General Feature Format (GFF) file as inputs to generate Allele Catalog datasets. The streamlined processing steps in the Allele Catalog pipeline consist of imputation, functional effect prediction, and aggregation of genotypic information and meta-information. The pipeline initially utilizes the Beagle imputation tool to impute the VCF file to fill the missing genotypes. The imputed VCF file is taken for functional effect predictions using the SnpEff tool to generate the functional effects and amino-acid change information. The fully imputed and annotated VCF file is processed by our in-house developed scripts to extract important functional effects from the file and restructure the genotype data to incorporate the meta-information, functional effect information, and imputation information in order to generate the Allele Catalog datasets. The output Allele Catalog datasets consist of genotypic information and meta-information such as improvement status, origins, accessions, genes, genetic variants, and predicted variant effects.

The Allele Catalog pipeline works on both standalone servers and high-performance computing clusters. Each task in the pipeline is scheduled and executed automatically in a parallel fashion. To use the pipeline, users only have to prepare the input files for the pipeline. The pipeline will process the input files by running through the same set of commands to generate the Allele Catalog datasets. The Allele Catalog datasets are tab-delimited datasets that can be easily stored and distributed. Therefore, the Allele Catalog datasets can be easily shared and processed by using any programming language in different research studies.

#### Datasets

In the Allele Catalog Tool development, we have selected 1066 unique soybean accessions from publicly available datasets such as Zhou302v2 [12], Liu304 [13], USB-15x, USB-40x [14], Soja [15], and MSMC [16] to generate the soybean VCF files and Allele Catalog datasets, which is also called Soy1066 data panel, and use it in the Allele Catalog Tool. The raw sequencing reads in FASTQ format and the Binary Alignment/Map (BAM) files of the 1066 accessions are collected from various sources like the National Center for Biotechnology Information (NCBI), European Nucleotide Archive (ENA), the Genome Sequence Archive (GSA) of the National Genomics Data Center (NGDC), and the Cyverse data store [17, 18]. The tools that are used in the data collection steps include the standard Linux GNU Wget program and the SRAtoolkit developed by the NCBI.

The downloaded 1066 samples are initially checked by using the FASTQC tool which is a quality control tool that can analyze the sequenced reads of FASTQ, SAM, and BAM files [19]. The 1066 samples are of good quality and no further trimming process is necessary. The SnakyVC variant calling pipeline built by our group was used to generate a VCF file from these 1066 samples and a Williams 82 version 2 reference genome (Wm82.a2.v1). The output VCF file has 1066 accessions and around 38 million positions (Table 1). The output VCF file is taken to the Allele Catalog pipeline along with a meta-information file and a soybean GFF file from Phytozome [20] to generate the soybean Allele Catalog datasets. At the end of the process, the Soy1066 data panel is successfully generated and can be utilized in the Soybean Allele Catalog Tool.

**Table 1:** A summary comparison of independent allele catalog tools developed for soybean, maize, and *Arabidopsis thaliana* provides details relevant to the underlying data for each tool

	**Soybean**	**Maize**	***Arabidopsis***
Name	Soy1066	MaizePanzeaAGPv3	Arabidopsis1135
Reference Genome	Wm82.a2.v1	AGPv3	TAIR10
Number of Accessions	1,066	1,208	1,135
Genome Size	978,495,272	2,067,864,162	119,667,750
Total Variant Positions	38,302,167	79,430,168	11,501,945
Total SNP Positions	32,524,427	74,306,694	11,462,068
Total Indel Positions	5,777,740	N/A	39,877
Mean of Missing Data Per Accession	3,412,030	40,704,160	1,987,800
Number of Genes	54,012	58,946	26,923
Number of Genes with Only Reference Allele	1,577	4,534	493
Number of Genes with Null Alleles	35,457	22,585	15,889
Number of Modifying Variants (SNPs and Indels)	618,501	1,093,495	1,129,470
Number of Null Variants (SNPs and Indels)	141,167	84,840	61,974
Mean of Number of Allele Positions Per Gene	40	62	129
Median of Number of Allele Positions Per Gene	22	43	91
Mean of Number of Alleles Per Gene	17	47	45
Median of Number of Alleles Per Gene	9	24	32

Table 1 Soybean, maize, and *Arabidopsis* Allele Catalog data information

Besides the Soy1066 data panel, our group also collected the *Arabidopsis* VCF files from the 1001 Genomes website (https://1001genomes.org/data/GMI-MPI/releases/v3.1/) [21] and the maize VCF files from the Panzea website (https://www.panzea.org/genotypes) [22]. The *Arabidopsis* VCF files have 1135 accessions and around 11 million positions reported with *Arabidopsis* TAIR10 reference genome coordinates, while the maize VCF files have 1208 accessions and around 79 million positions reported with the AGPv3 reference genome coordinates (Table 1). In the maize VCF files, the insertion and deletions are annotated as “ < INS > ” and “ < DEL > ” instead of the actual nucleotide sequence. Therefore, those indel positions are filtered from the VCF files and continue the analysis with approximately 74 million SNPs positions (Table 1). Both the *Arabidopsis* VCF files and the maize VCF files are treated as inputs to the Allele Catalog pipeline along with the respective meta-information files and GFF files to generate the Allele Catalog datasets for the Allele Catalog Tool.

The meta-information files that our group collected for soybean, *Arabidopsis*, and maize are mainly from supplemental files of datasets or published journal articles. The meta-information file for soybean is coming from existing supplemental files of Zhou302v2 [12], Liu304 [13], USB-15x, USB-40x [14], Soja [15], and MSMC [16], and the United States Department of Agriculture (USDA) Germplasm Resources Information Network (GRIN) database. The soybean meta-information file consists of accession, classification, improvement status, maturity group, country, and state information. Similarly, the meta-information for *Arabidopsis* is collected from the 1001 Genomes website (https://1001genomes.org/accessions.html) [21]. The *Arabidopsis* meta-information file consists of accession ID, accession name, latitude, longitude, country, state, and group information. The maize meta-information file is collected from the supplemental file of the Maize HapMapV3.2.1 [22]. It consists of accession, improvement status, dataset, and species information.

#### The Allele Catalog Tool

The Allele Catalog Tool is an interactive web-based Allele Catalog datasets visualization tool. The purpose of the Allele Catalog Tool is to provide data query, visualization, and download functions of the Allele Catalog datasets for users to browse the data on an interactive web interface and download the data for other purposes. The development of the Allele Catalog Tool includes a few components such as the database, the back-end processing code, and the front-end interfaces.

In the Allele Catalog Tool, we utilize the MySQL database to store our Allele Catalog datasets. Because of the large sizes of the datasets, making queries on the datasets can be slow. Therefore, the B + tree indexing method is used to speed up the queries. The queries of the Allele Catalog data are managed by the back-end code. The back-end code of the Allele Catalog Tool is developed using the PHP programming language. It processes users’ queries, gathers data from the database, processes the data, and returns the results to the front-end. The front-end code of the Allele Catalog Tool is written in HTML, CSS, and JavaScript. The front-end of the Allele Catalog Tool focuses on data rendering and user interactions. Having these three components working together, the Allele Catalog Tool can provide a good user experience.

Currently, the Allele Catalog Tool supports three species which are soybean, *Arabidopsis*, and maize. The Soybean Allele Catalog Tool is hosted on the SoyKB website [23–25] as one of the main tools. Apart from that, our research group also build the Allele Catalog Tool that supports maize and *Arabidopsis* on the KBCommons website [26, 27]. Hence, soybean, maize, and *Arabidopsis* researchers can benefit from the Allele Catalog Tool to advance their research.

## Results

### The Allele Catalog Tool

**Fig. 3:**
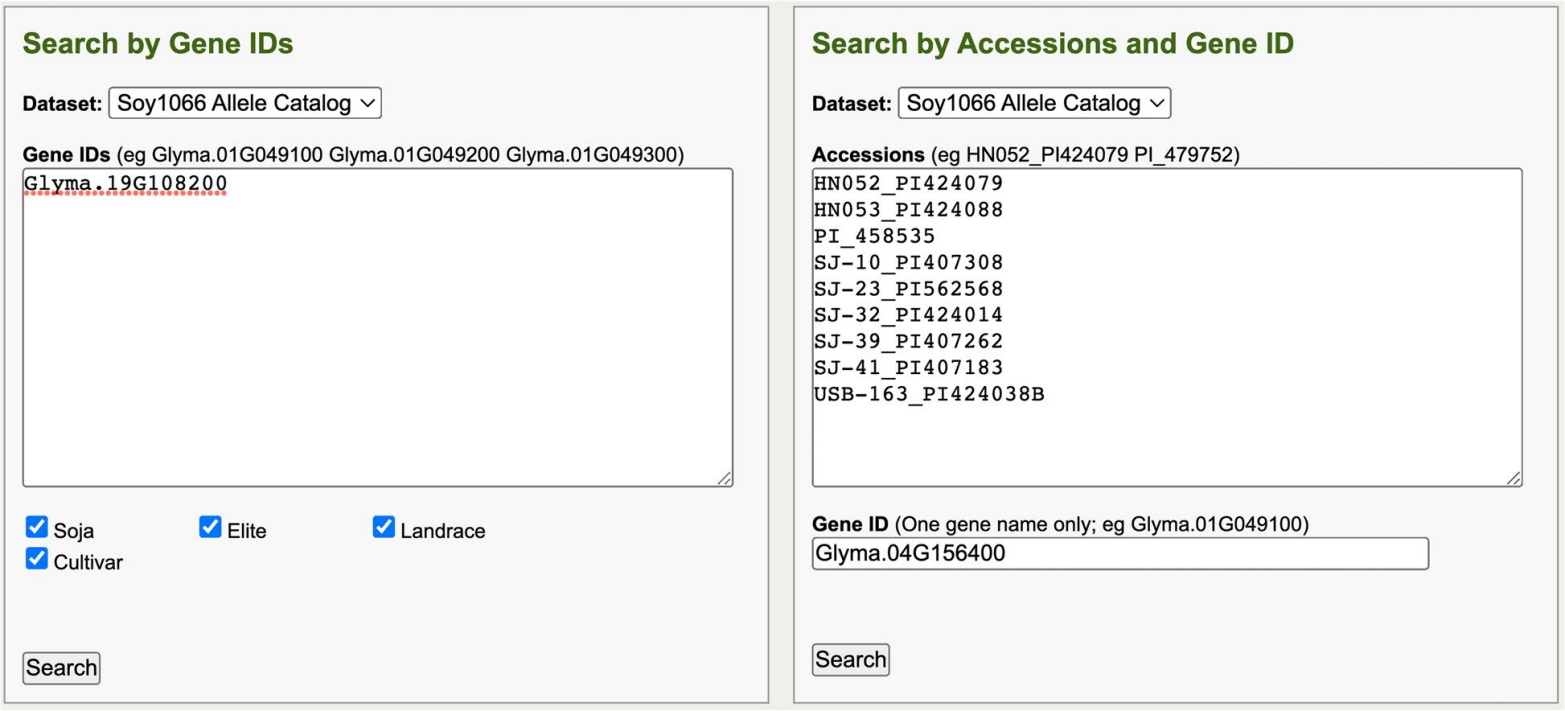
The Soybean Allele Catalog data query interface for users to query data by using a gene list (Search by Gene IDs box on left) or an accessions list with a single gene (Search by Accessions and Gene ID box on right)

The Allele Catalog Tool is a Gene IDs or accessions and gene ID-based search tool to explore allelic variation and frequency that relies on a pre-computed compilation of genomic sequence variants of a merged panel of re-sequenced accessions. The variant positions that result in modifying effects on genes are collated into alleles of every gene. The resulting visualization for each gene is a list of distinct alleles based on concatenated modifying changes compared to the reference annotation of the genome (the Allele Catalog). The frequencies of each of the alleles are reported along with a summary of categorical information about the accessions. The Allele Catalog data is produced from a reference genome sequence, a General Feature Format (GFF) gene annotation file, and WGS datasets containing categorical information and details about the accessions specific to each species. Categorical information provided with the accession names is summarized in a results table for each gene’s allele output. The basic Allele Catalog Tool is an online web-based tool that has user input query boxes for searching and opens a new webpage with the results (Fig. 3).

### The Allele Catalog query page for Searches by Gene IDs or by Accessions and Gene ID

In the Allele Catalog Tool, there are two options to perform queries (Fig. 3). One option is to input a list of genes of interest to perform a query. The second option is to input a list of accessions (one accession per line) and one gene to perform a query. To Search by Gene IDs, depending on the species, users can select from available datasets in the dropdown list, input one or more genes into the Gene IDs query box, and select to keep all data or select a subset of available categorical data types. The data subsets include the categorical information collected for each accession. For the Search by Accessions and Gene ID feature, users can enter one or more accession names in the Accessions query box, and input one gene in the Gene ID box. As an example for the Soybean Allele Catalog Tool searching with the Soy1066 Allele Catalog data, the soybean *FT5b* gene (*Glyma.19G108200*) [28] was entered into the Search by Gene IDs query box and the default for all available categorical subsets was selected (Fig. 3). Similarly, a list of soybean accessions and a single gene ID were entered into the Search by Accessions and Gene ID query box. The download Accession information button can be selected to provide a file with the available categorical data and meta-information with accession details for accessions in the datasets. To perform either query, users have to select the appropriate Search button below the respective query box (Fig. 3).

### Data visualization of results for Search by Gene IDs

After query entry and selection of the appropriate Search button, a new browser window opens with the data visualization of the results of the search. In the results, the Allele Catalog Tool renders the data queried by users in a tabular format with different alleles for each gene organized into rows. Each allele is defined by the unique combination of reference (Ref) or alternate genotypes at each of the observed variant positions in the gene. Each gene will have a table on the results page that presents allele frequency totals and can be subdivided for the categorical information present in the meta-information file. The two main parts of the results table are the frequency summary and the variant position genotype information, and these parts are separated by the Gene ID. For soybean, the frequency summary part of the results table includes the frequency counts of accessions with the allele from three categories of improvement status: *G. soja*, Landraces, and Elite with the summation of these categories plus any uncategorized accessions listed as the Total. The soybean Cultivar category is a separate categorical type in which we identified soybean accessions in the meta-information file that were developed and released as cultivars in North America. For each gene, the alleles are organized by Total frequency, with the most frequent being displayed at the top of the table. Users can check one or more boxes on either side of the results table to enlarge the text size of that allele.

**Fig. 4:**
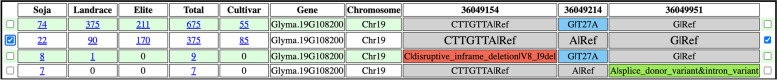
Data visualization for the Soybean Allele Catalog result window for Search by Gene IDs query for *Glyma.19G108200*

For *Glyma.19G108200*, there were three variant positions (an indel and two SNPs) with modifying effects predicted in the data that were listed by position number as the three column headings. In the allele table, predicted modifying changes are highlighted by color such that all reference positions are grey, severely deleterious mutations are red, conservative in-frame deletions or insertions are orange, missense mutations are blue, and predicted splice site changes are green; any other alternate genotypes that do not fit those categories are white. Reference genotypes only list the nucleotide(s) present; for alternate genotypes, the nucleotide(s) present and the predicted modifying change are given. For chromosome 19 nucleotide position 36,049,154 (Chr19: 36,049,154), the reference consisted of seven nucleotides, and the alternate had a six-base deletion (CTTGTTA vs. C) which resulted in a disruptive in-frame deletion and change in amino acid sequence (Fig. 4). For Chr19: 36,049,214, the A to G SNP resulted in a threonine to alanine missense mutation at amino acid 27 in the gene; and the Chr19: 36,049,951 SNP resulted in a predicted splice site mutation (Fig. 4). The most frequent allele (675 total accessions containing that allele) had the T27A missense mutation only, whereas the Williams 82 version 2 allele with the reference genotype at all positions (enlarged by clicking on the box to the side of the allele) was the second most frequent allele with 375 accessions containing that allele. The third most frequent allele had both the indel and the T27A missense mutation, and the fourth allele had a predicted splice site mutation only (Fig. 4). There are multiple options for downloading the data from the results pages as described below.

**Fig. 5:**
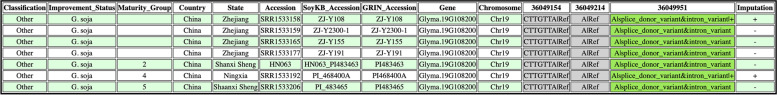
Data visualization for the Soybean Allele Catalog modal popup box for the results window for the seven *G. soja* accessions selected from the *Glyma.19G108200* frequency table that have the splice site allele. In the results window of the Allele Catalog, selecting one of the frequencies from the summary table triggers a modal popup box. The details from the meta-information file for each accession are provided along with an imputation data status indicator generally for the accession positions under the Imputation heading (+ for imputation;—for no imputation) and within genotype cells where an imputed genotype result is indicated as |+

In the results of the Search by Gene IDs for *Glyma.19G108200*, all of the numbers on the frequency tables (except zero) can be clicked on to trigger a modal popup feature that contains all of the detailed information from the accessions with that allele in that particular category. When the seven *G. soja* accessions are selected from the fourth (lowest total frequency/splice site) *Glyma.19G108200* allele results table, a modal popup box is the result (Fig. 5). This modal popup feature lists each accession and its associated categorical information along with other details from the meta-information (for soybean, Maturity Group, Country, State, and Accession). In addition, the allele table in the modal popup feature also consists of imputation information with the notation “ + ” as imputed and empty as unimputed for alleles (Fig. 5). Based on the imputation information of alleles, imputation at the gene level can also be summarized for each accession. Therefore, the imputation column in the table demonstrates the notation “ + ” to represent at least one imputed allele in a gene. Nonetheless, the notation “-” in the imputation column represents no imputed allele in a gene. In our example, accessions PI_468400A and ZJ-Y108 both contained imputed alleles on chromosome 19 position 36,049,951. Hence, the *Glyma.19G108200* gene of PI_468400A and ZJ-Y108 are considered genes with at least one imputed allele, and both accessions have a “ + ” notation in the imputation column. The other accessions, on the other hand, do not have any imputed alleles, so the “-” notation is used in the imputation column of those accessions. To dismiss the modal popup, users can click on the exit button in the top right corner of the modal.

### Data visualization of results for Search by Accessions and Gene ID

**Fig. 6:**
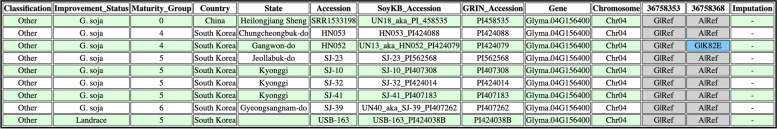
Data visualization for the Soybean Allele Catalog results window for Search by Accessions and Gene ID query for nine accessions and *Glyma.04G156400*

The Search by Accessions and Gene ID query essentially allows a list of accessions to be genotyped for a particular gene without frequency information. Nine soybean accessions and *Glyma.04G156400* were selected as the example for the Search by Accessions and Gene ID query (Fig. 3). After query entry and selection of the appropriate Search button, a new browser window opens with the data visualization of the results of the search (Fig. 6). These eight *G. soja* and one landrace accession ID were selected for having the indel and T27A missense allele of *FT5b Glyma.19G108200* (see Fig. 4), and *Glyma.04G156400* is the maturity gene *E1LA* [29]. The results of the search list the details for each accession from the meta-information file along with the Allele Catalog results for the variant positions in the selected gene (Fig. 6). The Search by Accessions and Gene ID results are formatted similarly to the modal popup feature from the Search by Gene IDs results with the exception that the allele information is unique for the queried gene for each accession. Accession HN052 (PI424079) had a K82E missense allele of *Glyma.04G156400*, while the other eight accessions contained the reference allele of *Glyma.04G156400*; none of the data was imputed (Fig. 6).

### Data download

In the Allele Catalog Tool, the queried data is downloadable by using the download buttons at the bottom of each table or each page (not shown). Under each table, there are two download buttons for users to download the data they queried. One of the buttons is for downloading the frequency table data of a specific gene, whereas another button is for downloading the Allele Catalog data of a specific gene. At the bottom of the page, there are two buttons for users to download the frequency tables of those queried genes and the Allele Catalog data of those queried genes. The data download function allows users to keep a copy of the data they queried and use the data for other research purposes.

### Case studies

#### Analyzing the frequency and distribution of null alleles of the soybean R gene for pigmentation and mining for novel alleles

**Fig. 7:**
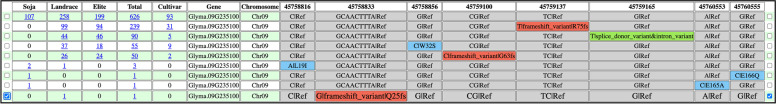
Data visualization for the Soybean Allele Catalog result window for Search by Gene IDs query for the *R* gene *Glyma.09G235100*

The genetics underlying soybean seed coat and hilum color involve multiple interacting genes controlling the biosynthesis of different classes of phenylpropanoid pathway-derived pigments. The classical *R* gene is an R2R3 MYB transcription factor that shifts pigment production from brown to black in appearance in some genetic contexts [30]. The food-grade market class of soybeans has a requirement for unpigmented (yellow) seed coats and hila, and *R* is one of the key components involved in yellow hilum and seed coat. Phenotyping for *R* is confounded by the effects of the other pigmentation genes, especially when the target is yellow seed coats and hila. Using the Soybean Allele Catalog tool with the Soy1066 default data and Search by Gene ID query for the *R* gene (*Glyma.09G235100*) revealed nine alleles, with the Williams 82 reference functional *R* allele having the highest frequency (Fig. 7). Consistent with the literature, there were two frameshift alleles, a splice site mutation allele, and a missense W32S allele present in the results table that had been previously described as nonfunctional *R* alleles of the gene [30]. The most frequent *R* allele in Cultivars was the R75fs frameshift (31 accessions) followed by the W32S missense allele (10 accessions); the splice site and alternate frameshift G63fs alleles were lower in frequency. Three new missense alleles and a novel frameshift allele were present with one to three accessions each (Fig. 7). When pigmentation phenotypes were extracted from the U.S. National Plant Germplasm System, with the exception of one accession without available seed coat or hilum color information, all of the accessions containing novel missense *Glyma.09G235100* alleles (L19I, E165A, and E166Q) were reported to have black seed coat and hilum colors. The single Q25fs frameshift accession, PI567258, was reported to have brown seed coat and hilum colors and represents a novel *R* allele.

#### Novel allelic variation in the maize florigen gene ZEA CENTRORADIALIS 8 (*ZCN8*)

**Fig. 8:**
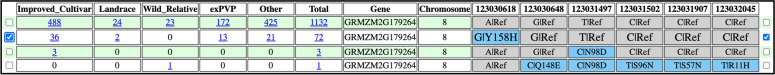
Data visualization for the Maize Allele Catalog result window for Search by Gene IDs query for the *ZCN8* gene *GRMZM2G179264*

The maize gene, *GRMZM2G179264*, ZEA CENTRORADIALIS 8 (*ZCN8*) is an FT-like gene that provides a *florigen function* to integrate photoperiod flowering signals and adapt to distinct climates [31–34]. Variants in the *ZCN8* promoter have previously been implicated in controlling gene expression, but coding sequence variation has not been described [32, 35]. Using *GRMZM2G179264* in the Maize Allele Catalog Search by Gene IDs query revealed a very high-frequency reference allele and only three other alleles (Fig. 8). We discovered a maize *ZCN8* allele with a Y158H missense mutation in 73 accessions (Fig. 8). The accessions containing the Y158H substitution consisted of early flowering accessions adapted to short-season environments as well as several tropical and semi-tropical accessions, so further research will be necessary to identify if the previously characterized *ZCN8* promoter variants are present and determine the effects of this missense allele in different genetic contexts.

#### Analysis of a low-frequency allele of *Arabidopsis* seed dormancy related gene delay of germination 1 (*DOG1*) in worldwide accessions

**Fig. 9:**
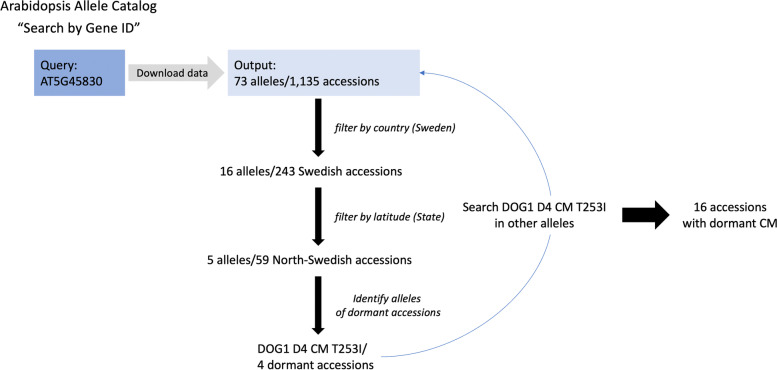
Scheme of *DOG1* gene (*AT5G45830*) analysis workflow. Geographical distribution of *DOG1 D4* allele was performed on downloaded data with filtering steps including filtering by description, alleles, and frequencies

Using the *Arabidopsis* Allele Catalog, researchers can analyze genes and alleles identified in their own studies in the worldwide context of 1,135 *Arabidopsis* accessions [21]. The frequency table data in the *Arabidopsis* Allele Catalog uses reduced population structure categorical information. In this case study, we analyzed the seed dormancy-related *DOG1* gene (*AT5G45830*) that can cause a delay in seed germination in *Arabidopsis thaliana* to find additional accessions that bear a rare allele that could be used in seed dormancy evolutionary studies. [36]. Seed dormancy is a complex trait that varies with geography, where high dormancy is associated with long, dry summers, and low dormancy is associated with short and wet summers [37–39]. A rare dormant allele (*D4*) was previously identified in *DOG1* specifically in North-Swedish accessions that possess high germination rates in general [40]. However, this allele was not studied thoroughly in the Kerdaffrec work due to its low allele frequency in the data set. Using the *Arabidopsis* Allele Catalog tool with the 1,135 *Arabidopsis* accessions default data and Search by Gene ID query for *AT5G45830* revealed 73 alleles (including *DOG1 D4*) composed of combinations of 59 modifying variant positions. *DOG1 D4* encompasses a single SNP at position Chr05: 18,590,289 [40] that leads to a threonine to isoleucine amino acid change (T253I). There are 17 accessions with the *DOG1 D4* allele. Further, there were two other alleles that contained the T253I (three accessions in total). To analyze the geographical distribution of the accessions with the *DOG1 D4* allele (Fig. 9), the downloaded results data were filtered for those with the country of origin in Sweden (*n* = 73). There were 16 alleles present in 243 Swedish accessions. These were further filtered based on latitude to North- (generally non-dormant) and South-accessions groups (potentially prone to dormancy). Five alleles were identified in North-Swedish accessions where one of them was the *DOG1 D4* that was found in four accessions. Obviously, *DOG1 D4* is rare in Sweden but not restricted to this country. The downloaded result of 73 alleles in *AT5G45830* was filtered for accessions with the T253I mutation. There were an additional 16 accessions from various geographical regions (Russia, Kazakhstan, Slovakia, Pakistan). It could be only speculated whether the geography of these accessions itself determines the possible dormant phenotype of these accessions and, further analyses would have to be performed to evaluate evolutionary aspects of the T253I emergence and possible dissemination of the *DOG1 D4* related alleles worldwide. However, the example of this *DOG1 D4* analysis demonstrated that the *Arabidopsis* Allele Catalog is a powerful and valuable source of information for a broad research community.

## Discussion

The Allele Catalog Tool mainly provides data query, visualization, and download functions for users to browse and utilize the Allele Catalog datasets. Using the Allele Catalog Tool, users can gain more understanding of functional genetics on gene and allele levels. All the tools have easy-to-use user interfaces that users can interact with. To access the Soybean Allele Catalog Tool, the best method is to go to the SoyKB website and select the Soybean Allele Catalog Tool under the tools section. On the other hand, to access the Allele Catalog Tool in the KBCommons for *Arabidopsis* and maize, users need to first select the organism and then they can find the Allele Catalog Tool under the tools section.

Although the development of the Allele Catalog Tool is completed, there are still a few challenges that can be addressed in future improvements to make it more efficient. The current Soy1066 data panel requires a lot of disk space for storage. In the future expansion of this data panel to create a larger data panel, it will cost a lot of computing resources and time for the variant calling and generating Allele Catalog datasets processes to complete. To solve this problem, the newly developed variant calling pipeline (SnakyVC) can still be utilized along with high-performance computing resources to generate a larger data panel in the future. SnakyVC pipeline is developed to provide reusable and efficient variant calling processing to make sure data consistency and reproducibility. Therefore, our research group believes that the continuous support and improvements of SnakyVC can ensure speed improvements and reduce storage space consumption, and at the same time, keep newly generated data consistent and reproducible. Apart from the problems in generating a larger data panel, collecting meta-information from various sources is also one of the difficult tasks. The meta-information file that is used in the Allele Catalog pipeline is usually collected from the supplemental tables of other publications and the United States Department of Agriculture (USDA) Germplasm Resources Information Network (GRIN) database. So, automating the process and selecting a few reliable sources for this information will be ideal in the future.

Considering the challenges our group could face when transferring the Allele Catalog concept to create a more generic version of the Allele Catalog Tool, we directly acquired pre-made maize and *Arabidopsis* VCF files from the Panzea website [22] and the 1001 genomes [21] to generate the Allele Catalog datasets and make the datasets available on the Allele Catalog Tool hosted on the KBCommons website. This allows us to skip the time-consuming variant calling processing step to make the concept easily expanded to other organisms. Apart from the challenges, since the current Allele Catalog Tool only focuses on the coding region, there are still many other mutation regions and related information that are not searchable and visualizable at the moment. The information includes promoter regions, transposable elements (TEs), and copy number variations which can also have an impact on the phenotype and is a useful piece of information for breeding and precision agriculture. Therefore, it is worth following the same ideas and methodologies to create a new toolset named Genomic Variation Explorer (GenVarX) to target the regions outside of coding regions and also expandable to support more organisms. Currently, the Genomic Variation Explorer supports both promoter regions and copy number variation analysis. It also covers organisms such as soybean, rice, and *Arabidopsis*. Thus, showing and describing the ideas and methodologies of the Allele Catalog Tool development are valued as they can assist readers and users to understand not only the Allele Catalog Tool but also the future products from our research group that can assist researchers in gaining a more comprehensive knowledge in plants.

## Conclusions

In the Allele Catalog Tool development, our research group has generated Allele Catalog datasets using the variant calling pipeline (SnakyVC) and the AlleleCatalog pipeline developed by us. Besides that, our research group also developed the Allele Catalog Tool and made the Allele Catalog datasets for soybean, *Arabidopsis*, and maize publicly available for querying, browsing, and downloading as part of the tool. Users who have genes of interest can use this tool to visualize alleles in genes. At the same time, users can also understand the functional effects and amino acid changes of SNPs and indels that occurred in genes. Using this tool, users can develop an understanding of allele diversity in genes and also understand clearly which accessions these variations may be coming from. Overall, this tool can be useful to users who are interested in the soybean gene and allele level discovery and incorporating that into their research work and breeding.

## Availability and requirements

Project Name: The Allele Catalog Tool.

Project Homepage:

- The Soybean Allele Catalog Tool: https://soykb.org/SoybeanAlleleCatalogTool/
- The Maize Allele Catalog Tool: https://kbcommons.org/system/tools/AlleleCatalogTool/Zmays
- The *Arabidopsis* Allele Catalog Tool: https://kbcommons.org/system/tools/AlleleCatalogTool/Athaliana

Pipeline Repositories:

- The variant calling pipeline (SnakyVC): https://github.com/yenon118/snakyVC
- The Allele Catalog pipeline (AlleleCatalog): https://github.com/yenon118/AlleleCatalog
  - In-house developed scripts are in the python folder inside the scripts folder

Operating Systems:

- The variant calling pipeline (SnakyVC): Linux operating system
- The Allele Catalog pipeline (AlleleCatalog): Linux operating system
- The Soybean Allele Catalog Tool: Platform independent
- The Maize Allele Catalog Tool: Platform independent
- The *Arabidopsis* Allele Catalog Tool: Platform independent

Programming Languages:

- Pipeline development: Python3 and Snakemake
- Web development: PHP, HTML, CSS, and JavaScript

Other Requirements:

- Pipeline development:
  - Python3 3.7.0 or higher
  - Snakemake 5.31.0 or higher
  - Python Data Analysis Library—Pandas 1.1.3 or higher
  - Burrows-Wheeler Aligner (BWA) 0.7.15 or higher
  - Genome Analysis Toolkit (GATK) 4.1.7.0 or higher
  - Samtools 1.10 or higher
  - High-Throughput Sequencing Library (HTSlib) 1.10 or higher
  - Beagle imputation tool 5.2 or higher
  - SnpEff functional effect prediction tool 5.1 or higher
- Web development:
  - PHP 8
- Web browsing:
  - Google Chrome (Recommended), Firefox, or Microsoft Edge

License:

- The variant calling pipeline (SnakyVC): MIT License
- The Allele Catalog pipeline (AlleleCatalog): MIT License
- The Soybean Allele Catalog Tool: MIT License
- The Maize Allele Catalog Tool: MIT License
- The *Arabidopsis* Allele Catalog Tool: MIT License

All the files that contain raw sequencing reads are from public sources [12–18]. The *Arabidopsis* and maize VCF files, and the meta-information files are from public data sources as well [20–22]. The soybean data panel (Soy1066) is accessible via https://soykb.org/public_data.php.

## Data Availability

1. The Soy1066 data panel: https://soykb.org/public_data.php 2. The Maize data panel: https://cbsusrv04.tc.cornell.edu/users/panzea/download.aspx?filegroupid=16 3. The *Arabidopsis* data panel: https://1001genomes.org/data/GMI-MPI/releases/v3.1/1001genomes_snp-short-indel_with_tair10_only_ACGTN.vcf.gz 4. The variant calling pipeline (SnakyVC): https://github.com/yenon118/snakyVC 5. The Allele Catalog pipeline (AlleleCatalog): https://github.com/yenon118/AlleleCatalog 6. The Soybean Allele Catalog Tool: https://soykb.org/SoybeanAlleleCatalogTool/ 7. The Maize Allele Catalog Tool: https://kbcommons.org/system/tools/AlleleCatalogTool/Zmays 8. The *Arabidopsis* Allele Catalog Tool: https://kbcommons.org/system/tools/AlleleCatalogTool/Athaliana 9. The Soy1066 accession list: Users can download the soybean accession list using the “Download Accession Information” button on the Soybean Allele Catalog Tool. 10. The Maize accession list: Users can download the maize accession list using the “Download Accession Information” button on the Maize Allele Catalog Tool. 11. The *Arabidopsis* accession list: Users can download the maize accession list using the “Download Accession Information” button on the Arabidopsis Allele Catalog Tool.

## Abbreviations

WGRS: Whole-genome re-sequenced data
GWAS: Genome-wide association studies
SNP: Single nucleotide polymorphism
Indels: Insertions and deletions
BWA: Burrows-Wheeler Aligner
GATK: Genome Analysis Toolkit
SAM: Sequence Alignment/ Map
BAM: Binary Alignment/ Map
GVCF: Genomic Variant Call Format
VCF: Variant Call Format
GFF: General Feature Format
NCBI: National Center for Biotechnology Information
ENA: European Nucleotide Archive
GSA: Genome Sequence Archive
NGDC: National Genomics Data Center
USDA: United States Department of Agriculture
GRIN: Germplasm Resources Information Network
TEs: Transposable elements
QTL: Quantitative Trait Loci
JSON: JavaScript Object Notation
